# Significance of Pretreatment Neutrophil-to-Lymphocyte Ratio in Mucoepidermoid Carcinoma of Pediatrics: A Multicenter Study

**DOI:** 10.3389/fped.2020.00096

**Published:** 2020-03-27

**Authors:** Hua Gao, Qing Gao, Jinlan Sun

**Affiliations:** ^1^Department of Oral Medicine, Central Hospital of Yingkou, Yingkou, China; ^2^Disease Control and Prevention Center, Shenyang, China

**Keywords:** salivary gland cancer, pediatric cancer, salivary cancer, neutrophil-to-lymphocyte ratio, mucoepidermoid carcinoma

## Abstract

**Background:** Our goal was to analyze the value of the pretreatment neutrophil-to-lymphocyte ratio (NLR) in the prognosis of pediatrics with parotid mucoepidermoid carcinoma (MEC).

**Methods:** Patients (≤ 18 years old) undergoing surgical treatment for primary parotid MEC were enrolled from multiple clinical centers retrospectively. The χ^2^-test was used to analyze the associations between clinicopathological variables and the NLR. The main study endpoints were recurrence-free survival (RFS) and disease-specific survival (DSS). The prognostic value of NLR was assessed by Kaplan–Meier method and Cox model analysis.

**Results:** There were 88 patients included in total, with mean NLR of 2.32 (range, 1.8–6.0). Histologic tumor grade and tumor stage were associated with the NLR significantly. The 10-year RFS rates were 98 and 81% for patients with an NLR < 2.32 and patients with an NLR ≥ 2.32, respectively, the difference was significant (*p* = 0.010). The 10-year DSS rate was 97 and 81% for patients with an NLR < 2.32 and patients with an NLR ≥ 2.32, respectively; the difference was not significant (*p* = 0.072). The independence of NLR in predicting the RFS was further confirmed in Cox model analysis.

**Conclusion:** The NLR significantly affects the prognosis in pediatrics with primary parotid MEC.

## Introduction

Parotid cancer is relatively uncommon in pediatrics (1–3), and the most common pathologic subtype is mucoepidermoid carcinoma (MEC), but because of the rarity of this disease, it is challenging to develop a consensus treatment. Moreover, there is some difference between pediatric and adult patients with parotid MEC regarding clinicopathological characteristics and disease prognosis (4), and reported predictors for recurrence and death in pediatrics include high tumor stage, high histological grade, and adverse pathologic characteristics (5–10).

There are important roles of interactions between the tumor cells and tumor microenvironment on cancer progression (11–13). The peripheral neutrophil-to-lymphocyte ratio (NLR) is an accurate and reliable inflammatory indicator. A number of previous authors have concluded that worse survival in solid cancers is possibly predicted by pretreatment high NLR (14, 15). However, whether there is similar phenomenon in parotid MEC remains unclear. Considering there is different immature lymphatic defense system in pediatrics, therefore, we aimed to assess the significance of the pretreatment NLR in the prognosis of pediatrics with parotid MEC.

## Materials and Methods

Our hospital institutional research committee had approved this study, and written informed consent was obtained from all the legal guardians for pediatric patients (≤ 18 years). Our research was performed based on the Declaration of Helsinki.

Pediatric patients (≤ 18 years old) with surgically treated primary parotid MEC were retrospectively enrolled between January 1991 and September 2018 in four hospitals in Liaoning. Related patient information regarding age, sex, TNM stage, operation, pathology, intraparotid node (IPN) metastasis, histologic tumor grade, recurrence, and death was extracted and analyzed. The histologic tumor grade and the tumor stage were defined according to the World health Organization 2017 classification and the American Joint Committee on Cancer seventh edition staging system, respectively. All pathologic sections were re-reviewed by at least two head and neck pathologists.

**Figure 1:**
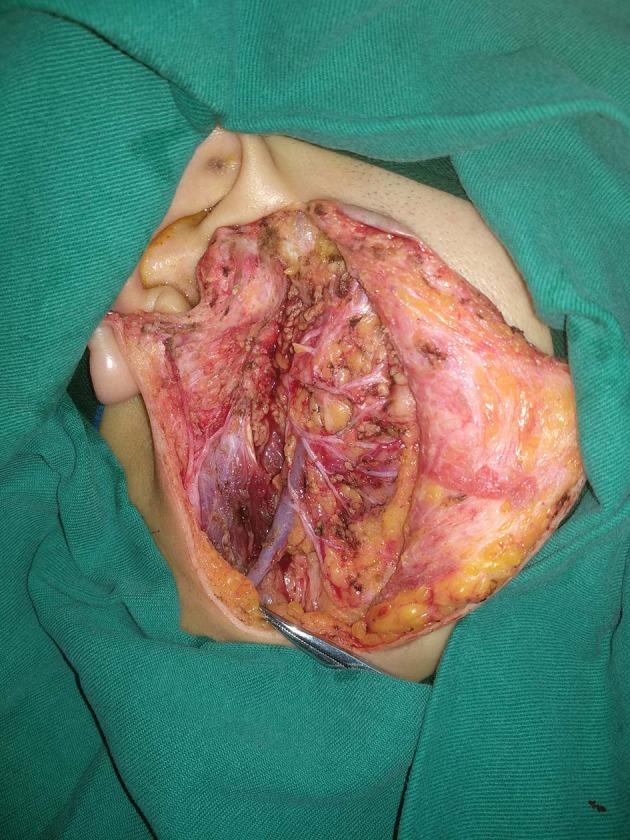
Superficial parotidectomy in a pediatric patient.

In our department, preoperative ultrasound, computed tomography, and/or magnetic resonance imaging examinations were routinely performed; fine-needle biopsy was selectively performed in the case of differential diagnosis from normal lymph nodes. Operation types of partial parotidectomy, superficial parotidectomy, and total parotidectomy were performed based on pathologic characteristics and the surgeon's experience (Figure 1). Facial nerve was tried to be preserved in every case. Adjuvant treatment was suggested if there was presence of positive margin, advanced tumor stage, and pathologic cervical lymph node metastasis.

The NLR was calculated within 2 weeks before the initial treatment by the ratio of the absolute neutrophil count to the absolute lymphocyte count (14–21). The previous reported cutoff values varied from 1.98 to 5 (14–20), and the standard cutoff value remains unknown. The cutoff value was defined as the mean value of the NLR in current study.

The associations between the NLR and the clinicopathological variables were analyzed using the χ^2^ test. The main study interests were the recurrence-free survival (RFS) and disease-specific survival (DSS). The survival time of RFS was calculated from the date of surgery to the date of first disease recurrence or the last follow-up, and the survival time of DSS was calculated from the date of surgery to the date of cancer-caused death or the last follow-up. The RFS and DSS rates were analyzed by the Kaplan–Meier method. The factors that were significant in univariate analysis were then analyzed in the Cox model to find out the independent prognostic factors. All reported *p*-values were two-sided, and a *p* < 0.05 was considered to be significant; all statistical analyses were performed by SPSS 20.0, IBM, USA.

## Results

There were 88 patients (53 female and 35 male patients) in total enrolled in the study with mean age of 14.2 years (range, 6–18 years), including 23 patients from the Affiliated First Hospital of China Medical University, 18 patients from the Affiliated Stomatology Hospital of China Medical University, 42 patients from the Affiliated Shengjing Hospital of China Medical University, and 5 patients from the Central Hospital of Yingkou. A history of blood malignancy was reported in 12 patients, and the mean duration between the diagnosis of previous blood malignancy and the diagnosis of parotid MEC was 8.0 years, with a range from 5 to 11 years. Thirty-five patients had T1 disease, 38 patients had T2 disease, 10 patients had T3 disease, and 5 patients had T4 disease. Operations of superficial parotidectomy, partial parotidectomy, and total parotidectomy were performed in 26, 13, and 49 patients, respectively. Six patients had facial nerve branches resected because of tumor invasion. Eighty patients had a clear margin. Neck dissection was performed in 23 patients, of whom 10 patients had metastatic neck lymph nodes; the mean number of positive nodes was 1.1, with a range from 1 to 3. Lymphovascular invasion and perineural invasion were presented in 8 and 10 patients, respectively. Low-, moderate-, and high-grade diseases were noted in 67, 14, and 7 patients, respectively.

A total of 77 patients had IPN information, and 16 patients had IPN metastases. The mean positive lymph nodes diameter was 0.8 cm with a range from 0.4 to 1.9 cm. The mean number of positive IPNs was 1.1, with a range from 1 to 3.

The NLR varied from 1.8 to 6.0 with mean value of 2.32. Table 1 presents the associations between clinicopathological variables and NLR. Histologic tumor grade and tumor stage were associated with the NLR significantly (both *p* < 0.05) (Table 1).

**Table 1:** Association between neutrophil-to-lymphocyte ratio and clinical pathologic variables.

**Variables**	**Neutrophil-to-lymphocyte ratio**		***p***
	**<2.32 (*n* = 48)**	**≥2.32 (*n* = 40)**	
Age			
<14 y	12 (25.0%)	8 (20.0%)	
≥14 y	36 (75.0%)	32 (80.0%)	0.577
Sex			
Female	30 (62.5%)	23 (57.5%)	
Male	18 (37.5%)	17 (42.5%)	0.633
Tumor stage			
T1 + T2	44 (91.7%)	29 (72.5%)	
T3 + T4	4 (8.3%)	11 (27.5%)	0.023
Node stage			
N0	45 (93.8%)	31 (77.5%)	
N+	3 (6.2%)	7 (22.5%)	0.099
Disease stage			
I + II	42 (87.5%)	26 (65.0%)	
III + IV	6 (12.5%)	14 (35.0%)	0.012
Perineural invasion			
Positive	5 (10.4%)	5 (12.5%)	
Negative	43 (89.6%)	35 (87.5%)	0.759
Lymphovascular invasion			
Positive	3 (6.3%)	5 (12.5%)	
Negative	45 (93.7%)	35 (87.5%)	0.460
Intraparotid node metastasis			
Positive	5 (10.4%)	11 (27.5%)	
Negative	31 (89.6%)	30 (72.5%)	0.163
Histologic tumor grade			
Low	39 (81.3%)	28 (70.0%)	
Intermediate + high	9 (18.7%)	12 (30.0%)	0.218
Blood malignancy history			
Yes	5 (10.4%)	7 (17.5%)	
No	43 (89.6%)	33 (82.5%)	0.335

*Gray values indicates the factor is significant*.

**Figure 2:**
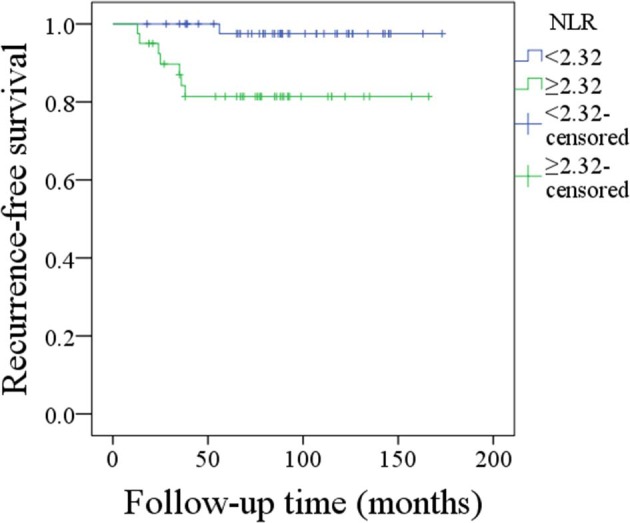
Recurrence-free survival of patients with different neutrophil-to-lymphocyte ratios (*p* = 0.010).

During our follow-up with mean time of 88.4 (range, 18–173) months, 21 patients received adjuvant radiotherapy, and 4 patients received adjuvant chemotherapy. Recurrence occurred in 8 patients: 4 cases locally, 2 cases locoregionally, 1 case regionally, and 1 case distantly. The 10-year RFS rates were 98 and 81% for patients with an NLR < 2.32 and patients with an NLR ≥ 2.32, respectively, the difference was significant (*p* = 0.010; Figure 2). Cox proportional hazards model analysis reported the independence of the NLR in affecting the RFS (Table 2).

**Table 2:** Predictors for recurrence-free survival in pediatric patients with parotid mucoepidermoid carcinoma.

**Variables**	**Univariate analysis**	**Multivariate analysis**	
		**HR (95% CI)**	***p***
Age (≤ 14 vs. >14)	0.321		
Sex	0.155		
Nerve invasion	0.099		
Lymphovascular invasion	0.541		
Radiotherapy	0.222		
Margin status	0.077		
Node stage (N0 vs. N+)	0.561		
Tumor stage (T1 + T2 vs. T3 + T4)	<0.001	2.122 (0.973–5.223)	0.057
Disease stage (I + II vs. III + IV)	0.003	3.123 (1.339–8.264)	<0.001
Grade (low vs. intermediate + high)	<0.001	1.596 (1.096–4.123)	0.005
Blood malignancy history	<0.001	1.639 (1.002–3.885)	0.019
Intraparotid node metastasis	<0.001	2.445 (1.339–5.697)	0.003
Resection extent (TP vs. PP + SP^*^)	0.050		
NLR (<2.32 vs. ≥2.32)	0.010	1.882 (1.021–3.162)	0.037

* *TP, total parotidectomy; PP, partial parotidectomy; SP, superficial parotidectomy*.

*Gray values indicates the factor is significant*.

**Figure 3:**
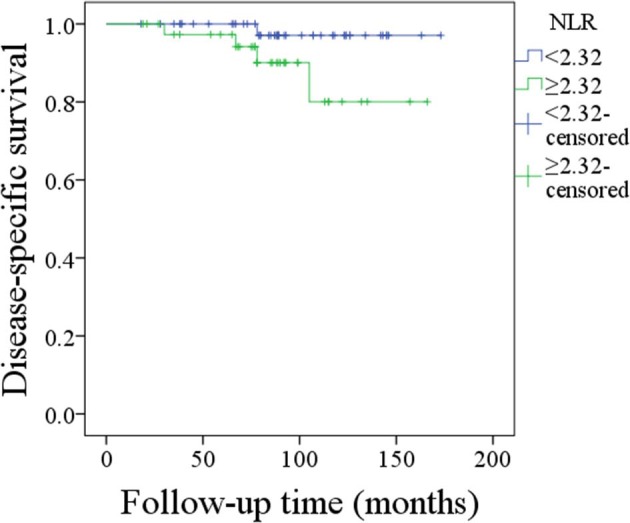
Disease-specific survival of patients with different neutrophil-to-lymphocyte ratios (*p* = 0.072).

Disease-caused death occurred in 5 patients, and the 10-year overall DSS rate was 91%. The 10-year DSS rate was 97% and 81% for patients with an NLR < 2.32 and patients with an NLR ≥ 2.32, respectively, the difference was not significant (*p* = 0.072; Figure 3). Cox proportional hazards model analysis reported the independence of histologic tumor grade, disease stage, malignancy history, and IPN metastasis status in predicting the DSS (Table 3).

**Table 3:** Predictors for disease-specific survival in pediatric patients with parotid mucoepidermoid carcinoma.

**Variables**	**Univariate analysis**	**Multivariate analysis**	
		**HR (95% CI)**	***p***
Age (≤ 14 vs. >14)	0.339		
Sex	0.842		
Nerve invasion	0.083		
Lymphovascular invasion	0.133		
Radiotherapy	0.384		
Margin status	0.194		
Node stage (N0 vs. N+)	0.287		
Tumor stage (T1 + T2 vs. T3 + T4)	<0.001	1.401 (0.456–8.112)	0.321
Disease stage (I + II vs. III + IV)	0.009	2.669 (1.269–6.716)	0.005
Grade (low vs. intermediate + high)	<0.001	3.172 (1.228–7.654)	0.002
Blood malignancy history	0.003	1.555 (1.011–2.682)	0.011
Intraparotid node metastasis	<0.001	2.146 (1.358–6.012)	0.006
Resection extent (TP vs. PP + SP^*^)	0.511		
NLR (<2.32 vs. ≥2.32)	0.072		

* *TP, total parotidectomy; PP, partial parotidectomy; SP, superficial parotidectomy*.

*Gray values indicates the factor is significant*.

## Discussion

The most important finding in the current study was that histologic tumor grade and tumor stage were associated with the NLR significantly, and also an NLR ≥ 2.32 meant worse disease prognosis would be expected. The finding could benefit all the pediatric surgeons and head and neck surgeons in better controlling the cancer.

The usefulness of the NLR was only previously analyzed in allergic conditions, inflammatory disorders, and infectious diseases in children (22–24). Vasquez et al. (25) first found that an NLR >2 was an independent indicator of worse overall survival in pediatric sarcomas; significant associations were noted between a high NLR and metastatic disease or poor histological response. We are the first to evaluate the significance of the NLR in pediatric patients with parotid MEC, and a high NLR predicted a decreased RFS rate in multivariate analysis. With regard to adult patients, there were only two articles focusing on this question (16, 17). Damar et al. (16) found that compared with patients with malignant salivary gland tumor the NLR was significantly lower in patients with benign salivary tumors, and the NLR significantly differed with histologic tumor grade. The finding was consistent with ours; it was also described that there was significant association between the NLR and disease stage. Kawakita et al. (17) described that compared to salivary duct carcinoma patients with baseline NLR, an NLR > 2.5 meant a nearly two-fold increase in the risk of death in overall survival.

The exact mechanisms for explaining the associations between the NLR and clinicopathologic variables or prognosis remain unclear, but based on previous evidence some possible explanations can be inferred. The pretreatment NLR is an indicator for the immune system and systemic inflammation. Several angiogenic factors and cytokines, which play an important role in promoting tumor development, can be generated by neutrophils (26). On the other hand, hematological markers might be associated with cancer cachexia; it carried poor survival (17, 27). In contrast, lymphocytes are associated with immune surveillance and play an important role of eliminating cancer cells (28). Therefore, the NLR might act as a predictor for the prognosis.

The issue of IPN metastasis in pediatric parotid MEC was rarely assessed. We first describe that IPN metastasis predicts worse RFS and DSS. There were similar findings in adults (29–32). Lim et al. (29) might have been the first to identify patients with cN0 neck disease, but their patients with IPN metastasis were more likely to develop locoregional recurrence than their patients who did not have IPN metastasis. Klussmann et al. (30) noted that there was an additional significant risk for tumor recurrence related to the involvement of the IPNs in 55 patients with pN+ disease. Nisa et al. (31) reported that in patients with IPN metastasis decreased disease-free survival could be foreseen. Therefore, IPN metastasis was associated with worse RFS and DSS in pediatrics as well as adults. The underlying mechanism might be that first there were superficial and deep lobe lymph nodes in parotid; recurrence would be aroused owing to unresected positive IPN tissue left by un-total parotidectomy. Second, the parotid lymph nodes are not part of any neck lymph node levels, but the IPNs might act as a predictor for neck lymph node metastasis.

We noted a history of malignancy-predicted worse RFS and DSS. A secondary parotid malignancy in pediatric patients has been reported by case reports (33), and owing to the extreme rarity, the prognosis of those cancers remains unknown. Védrine et al. (5) reported there was no significant difference regarding pathologic tumor grade and tumor location between patients with primary and secondary parotid MEC, but patients with secondary parotid MEC had less advanced stage than patients with primary disease. However, the two groups had similar overall survival, DSS, and disease-free survival. There were only five deaths in the current study; worse DSS was noted in patients with a history of malignancy. Previous chemotherapy, which had a significant adverse impact on the lymphatic system, might be partially responsible.

The prognostic significance of CRTC1/3-MAML2 fusion in MEC has been analyzed. Okumura et al. (34) reported that in early-stage MEC patients positive for CRTC1/3-MAML2 fusions, an excellent prognosis may be achieved without adjuvant radiotherapy when the tumors are completely resected without tumor spillage. However, Birkeland et al. (35) found a high rate of CRTC1/3-MAML2 gene fusions in a large cohort of MEC patients, but the authors did not note any correlation between fusion status and tumor grade or survival. Moreover, whether a similar phenomenon occurs in pediatric patients remains unclear, and more high-quality studies are required to illustrate clearly.

Limitations in the current study must be stated: first, the statistical power was decreased by our inherent bias in retrospective study and our relatively small sample size. Second, neutrophil and lymphocyte counts are easily influenced by infections or inflammation; they are nonspecific parameters. Third, because of differences in diagnostic ability by the different pathologists in four hospitals, there might be undetected IPN metastases.

## Conclusion

In summary, the pretreatment NLR is significantly associated with survival in pediatric patients with parotid MEC.

## Data Availability Statement

All datasets generated for this study are included in the article/supplementary material.

## Ethics Statement

The Zhengzhou University institutional research committee approved our study, and all of the legal guardians, including parents, provided written informed consent for any patient under the age of 18 years old. This study was conducted in accordance with the Declaration of Helsinki. All methods were performed in accordance with the relevant guidelines and regulations.

## Author Contributions

All authors make significant contribution in data collection, analysis, and manuscript writing and revision.

### Conflict of Interest

The authors declare that the research was conducted in the absence of any commercial or financial relationships that could be construed as a potential conflict of interest.

## Abbreviations

MEC: mucoepidermoid carcinoma
IPN: intraparotid node
NLR: neutrophil-to-lymphocyte ratio
RFS: recurrence-free survival
DSS: disease-specific survival.
